# Low-Density Lipoprotein Receptor Related protein-1 (LRP1)-Dependent Cell Signaling Promotes Neurotrophic Activity in Embryonic Sensory Neurons

**DOI:** 10.1371/journal.pone.0075497

**Published:** 2013-09-23

**Authors:** Kazuyo Yamauchi, Tomonori Yamauchi, Elisabetta Mantuano, Kenichi Murakami, Kenneth Henry, Kazuhisa Takahashi, Wendy Marie Campana

**Affiliations:** 1 Department of Anesthesiology, School of Medicine, University of California San Diego, La Jolla, California, United States of America; 2 Department of Orthopedic Surgery, Graduate School of Medicine, Chiba University, Chiba, Japan; 3 Program in Neurosciences, University of California San Diego, La Jolla, California, United States of America; Biogen Idec, United States of America

## Abstract

Developing sensory neurons require neurotrophic support for survival, neurite outgrowth and myelination. The low-density lipoprotein receptor-related protein-1 (LRP1) transactivates Trk receptors and thereby functions as a putative neurotrophin. Herein, we show that LRP1 is abundantly expressed in developing dorsal root ganglia (DRG) and that LRP1-dependent cell signaling supports survival, neurite extension and receptivity to Schwann cells even in the absence of neurotrophins. Cultured embryonic DRG neurons (E15) were treated with previously characterized LRP1 ligands, LRP1-receptor binding domain of α2-macroglobulin (RBD), hemopexin domain of MMP-9 (PEX) or controls (GST) for two weeks. These structurally diverse LRP1 ligands significantly activated and sustained extracellular signal-regulated kinases (ERK1/2) 5-fold (p<0.05), increased expression of growth-associated protein-43(GAP43) 15-fold (P<0.01), and increased neurite outgrowth 20-fold (P<0.01). Primary sensory neurons treated with LRP1 ligands survived > 2 weeks *in vitro*, to an extent equaling NGF, a finding associated with canonical signaling mechanisms and blockade of caspase-3 cleavage. LRP1 ligand-induced survival and sprouting were blocked by co-incubation with the LRP1 antagonist, receptor associated protein (RAP), whereas RAP had no effect on NGF-induced activity. Site directed mutagenesis of the LRP1 ligand, RBD, in which Lys^1370^ and Lys^1374^ are converted to alanine to preclude LRP1 binding, were ineffective in promoting cell signaling, survival or inducing neurite extension in primary sensory neurons, confirming LRP1 specificity. Furthermore, LRP1-induced neurite sprouting was mediated by Src-family kinase (SFK) activation, suggesting transactivation of Trk receptors. Co-cultures of primary embryonic neurons and Schwann cells showed that LRP1 agonists promoted axonal receptivity to myelination to Schwann cells. Collectively, these findings identify LRP1 as a novel and perhaps essential trophic molecule for sensory neuronal survival and development.

## Introduction

Embryonic sensory neurons undergo substantial programmed cell death during development [1] to ensure balance between neuronal numbers and the sizes of their target areas. During neurogenesis in the rat, (E10-E13) neuronal precursors, post mitotic sensory neurons and fully differentiated proprioceptors are dependent on locally synthesized BDNF and NT-3, and to a lesser extent NGF, but then switch their neurotrophin survival requirements to NGF by E15 [2,3]. Accordingly, levels of TrkA and p75 mRNA and protein are markedly elevated at day E15 [4]. Thus, developing sensory neurons are differentially regulated by various neurotrophins to survive and innervate target tissues. Switching neurotrophic support during development is tightly regulated by cell signaling mechanisms, many of which are currently under investigation.

More recent reports have suggested a potential role for a member of the LDL gene receptor family member, the low-density lipoprotein (LDL) receptor-related protein-1 (LRP1), in neural development and regeneration. LRP1 is the most multifunctional member of the LDL receptor gene family [5]. The mature receptor is derived from a large (600 kDa) two chain transmembrane receptor that is proteolytically processed upon furin cleavage. The processed form of the receptor includes an 85 kDa fragment that contains the intracellular and transmembrane domain and a 515 kDa extracellular domain that is capable of binding multiple LRP1 ligands [6]. Prior to its discovery in the PNS [7], LRP1 has been implicated in two main biological functions: endocytosis of its numerous ligands and modulation of cell signaling pathways. Structurally diverse ligands including tissue-type plasminogen activator (tPA), matrix metalloproteinase-9 (MMP-9), and activated α_2_-macroglobulin have been shown to activate LRP1-dependent cell signaling [5,8,9,10,11]. More recently, LRP1 ligands were shown to transactivate Trk receptors in a Src-family kinase dependent manner [12].

Global deletion of the LRP1 gene in the mouse highlights the biological importance of this receptor and reveals a critical but yet undefined role in development [13]. Furthermore, LRP1 knockouts in forebrain neurons facilitates synaptic loss and neurodegeneration [14], whereas LRP1 agonists promote axonal growth after dorsal column lesions [15]. These findings potentially identify a candidate mechanism for broad and essential actions of LRP1 in sensory neuronal survival and function.

Herein, we demonstrate that recombinant proteins, produced specifically to augment LRP1 signaling without off-target effects [11,16], induced primary embryonic sensory neuron survival, neurite outgrowth and myelination by blocking caspase-3 cleavage and sustaining ERK1/2 activation even in the complete absence of NGF. Furthermore, when LRP1-dependent cell signaling is blocked by competitive antagonists or genetic mutation, ERK1/2 activation, neurite outgrowth, and neuronal survival are inhibited. Our findings suggest that LRP1 is a novel trophic molecule for sensory neuronal survival and development.

## Material and Methods

### Ethics Statement

All animal procedures were approved by the Institutional Animal Care and Use Committee of the University of California, San Diego. In accordance with OLAW guidelines, rats were euthanized in a non-pre-charged container with 100% carbon dioxide introduced into the chamber and volume is measured with a flow meter attached to the carbon dioxide canister. Animals remain in the chamber a minimum for 5 min. These methods bring rapid death and results in minimal distress to the animal. This method is consistent with the recommendations of the Panel on Euthanasia of the American Veterinary Medical Association.

### Reagents

Glutathione-S-transferase (GST) fusion proteins that directly bind LRP1 and act as LRP1 agonists are expressed in bacteria, purified to homogeneity and characterized as previously described [11,16]. The fusion proteins include: 1) the 18kDA **R**eceptor **B**inding **D**omain of α2-macroglobulin (α_2_M), henceforth referred to as RBD [11,17,18,19], which encompasses amino acids 1242 to 1451 of the mature α_2_M structure and is an LRP1 agonist; and 2) the 18 kDa LRP1 binding domain of matrix metalloprotease-9, the hemopexin domain, henceforth referred to as MMP-9-PEX [20], which includes amino acids 514 to 704 of mature MMP-9 and is an LRP1 agonist. We also prepared fusion proteins that preclude LRP1 binding. These include 1) a well established LRP1 competitive antagonist, consisting of the 39 kDa Receptor Associated Protein (RAP) expressed as previously described [21] and a mutated form of RBD, that changes two lysines (1370,1374) to alanines [11,17]. As a control, we expressed GST in bacteria transformed with the empty vector, pGEX-2T. After purification, the RBD, PEX, RAP and GST were subjected to chromatography on Detoxi-Gel endotoxin-removing columns (Pierce). Murine NGF-β was purchased from Sigma (St. Louis, MO). The Src-family kinase selective inhibitor (SFK), (PP2); 3-(4-chlorophenyl) 1-(1,1-dimethyl)-H-pyrazolo[3,-d] pyrimidine-4-amine), was purchased from Calbiochem (San Diego, CA).

### Primary embryonic DRG neurons

Primary cultures of DRG neurons were prepared from embryo day 15 (E15) Sprague-Dawley rats as described [22,23] with some modifications [24]. National Institutes of Health and Institutional Animal Use and Safety Committee guidelines for laboratory animal care and safety were strictly followed for all animal use. DRG cells were isolated from tissue by mechanical dissociation in a solution that contained trypsin (1 mg/ml) for 15 min at 37°C. The cells were plated on glass coverslips coated with collagen type I (25 µg/ml) at a density of 120,000 cells/10 cm^2^ (6-well plate) and cultured in Ultraculture media with 2 mM L-glutamine (Mediatech), 1% penicillin-streptomycin (100 µg/ml; GIBCO) under a 5% CO_2_ atmosphere at 37°C. The DRG cells were then treated every 2 days with 10 µM uridine and 10 µM 5-fluoro-2’-deoxyuridine to remove all non-neural cells. Cells were treated with RBD (100 nM), MMP-9-PEX (100 nM), NGF (50 ng/ml) or GST (100 nM) immediately after plating DRG cells and subsequently every other day in fresh media. In some experiments, DRG cells were pretreated for 30 min prior to the addition of LRP1 ligands with or without GST-RAP (100 nM) or PP2 (1 µM). After two weeks, cell viability in each well was assessed by Trypan Blue exclusion.

### Primary Schwann cell cultures

Schwann cells were isolated from the sciatic nerve of 1-day-old Sprague-Dawley rats as previously described [19,25]. Final preparations contained at least 95% Schwann cells, as determined by immunofluorescence for S100, which is a specific Schwann cell marker. Primary cultures of Schwann cells were maintained in DMEM containing 10% FBS (HyClone Laboratories, Logan, UT), 1% penicillin-streptomycin, bovine pituitary extract (21 µg/ml; Clonetics), and 4 µM forskolin (Calbiochem) at 37°C under humidified 5.0% CO_2_.

### Schwann cell and embryonic DRG co-cultures

We adapted the co-culture systems previously developed [23,26]. DRG neurons were cultured in RBD (100 nM), GST (100 nM) or NGF (50 ng/ml) for 2 weeks. Schwann cells (600,000 cells/cm^2^) were subsequently added to established primary DRG neurons cultures. Cells were subsequently co-cultured for 48 hours in Ultraculture media supplemented with 10% FBS, 2 mM L-glutamine, 1% penicillin-streptomycin at 37°C under humidified 5.0% CO_2_.

### RNA isolation and real-time qPCR

RNA was extracted from cultured embryonic neurons (GAP-43; Applied Biosystems, Inc.) or co-cultures of neurons and Schwann cells (P0 and MAG; Applied Bio Systems, Inc.). DNA-free total RNA was extracted using Trizol (Invitrogen) and treated with Turbo DNA-free DNase. cDNA was synthesized using the ProSTAR first-strand RT-PCR kit (Stratagene). The one-step program included 2 min at 50°C, 10 min at 95°C, followed by 40 cycles of 95°C for 15 s; 60°C for 1 min, using an ABI 7300 instrument. Rat cyclophilin mRNA was measured in each sample as a housekeeping gene [7]. Samples without cDNA were analyzed as “no-template” controls. mRNA levels were measured in duplicate, in four separate experiments, and normalized against the Ct value for the housekeeping gene. For the GST group, several wells were pooled to yield adequate total RNA levels.

### Immunofluorescence and microscopy

For immunofluorescence studies, cells were plated on glass coverslips and then fixed in absolute methanol. Fixed cells were permeabilized in 0.2% Triton X-100 for 5 min and nonspecific binding was blocked with 1% bovine serum albumin for 30 min at 37°C. Primary antibodies included cleaved caspase-3 (1:500; Cell Signaling) and monoclonal antibody neuronal Class III β-tubulin (Tuj1; 1:500; Covance) that were incubated overnight at 4°C. The cells were then rinsed in PBS and incubated with secondary antibodies conjugated to either Alexa 594 conjugate (red) or Alexa 488 (green) for 30 min at 22°C. In some cases, dual labeling with cleaved caspase-3 and Tuj1 was performed as described [15]. Sections were mounted on slides using Pro-long Gold with 4,6-diamidino-2-phenylindole (DAPI) (Invitrogen) to identify nuclei. Sections without primary antibody served as a non-specific control. Imaging was performed using an Olympus MVX10 (Tokyo, Japan) fluorescence microscope and OpenLab software. Neurons labeled by Tuj1 a specific marker of neurons were imaged manually x100 magnification, and the longest neurite length per cell was measured in a minimum of 60-80 neurons/well using imageJ. Quantification was performed in a blinded manner.

### Phosphorylation studies and immunoblots

PC12 cells or primary cultures of embryonic DRG cells cultured in NGF (50 ng/ml) for 2 weeks were incubated in serum free media (4 or 1 h, respectively), cells were treated with RBD (100 nM), NGF (50 ng/ml), or GST (100 nM) for 0, 10, 30, 60 or 90 min. In some experiments, GST-RAP was incubated with the cells for 15 min prior to the addition of LRP1 agonists and NGF. Cells were then rinsed twice with ice-cold PBS and lysed in RIPA buffer (PBS with 1% Triton X-100, 0.5% sodium deoxycholate, 0.1% SDS, proteinase inhibitor mixture and sodium orthovanadate) as previously described [27]. The protein concentration in tissue extracts was determined by bicinchoninic acid assay. An equivalent amount of cellular protein (50 µg per lane) was subjected to 10% SDS-PAGE and electrotransferred to nitrocellulose membranes. Membranes were blocked with 5% nonfat dry milk in 10 mM Tris-HCl, 150 mM NaCl, pH 7.4, with 0.1% Tween 20 and incubated with primary antibodies (anti-pERK1/2, anti-pAKT, anti-tERK1/2; Cell Signaling, and anti-LRP1 and anti-tubulin; Sigma) diluted in TBS containing 5% BSA overnight at 4°C. The membranes were washed and followed by incubation with horseradish peroxidase-conjugated secondary antibodies for 1 h at room temperature. Immunoblots were developed using enhanced chemiluminescence ECL Plus™ (GE Healthcare) and HyBlot CL Autoradiography film (Denville Scientific Inc.). Blots were scanned (Canoscan) and densitometry was performed (NIH Image).

### Statistical analysis

Neurite extension, qPCR, and immunoblots were subjected to one-way ANOVA. Tukey’s or Student’s Neuman Keuls *post hoc* analysis was used to assess the differences between treatment groups. Data were analyzed with PRISM (La Jolla, CA).

## Results

### RBD activates LRP1-dependent cell signaling that is sustained in PC12 and primary embryonic DRG neurons

**Figure 1:**
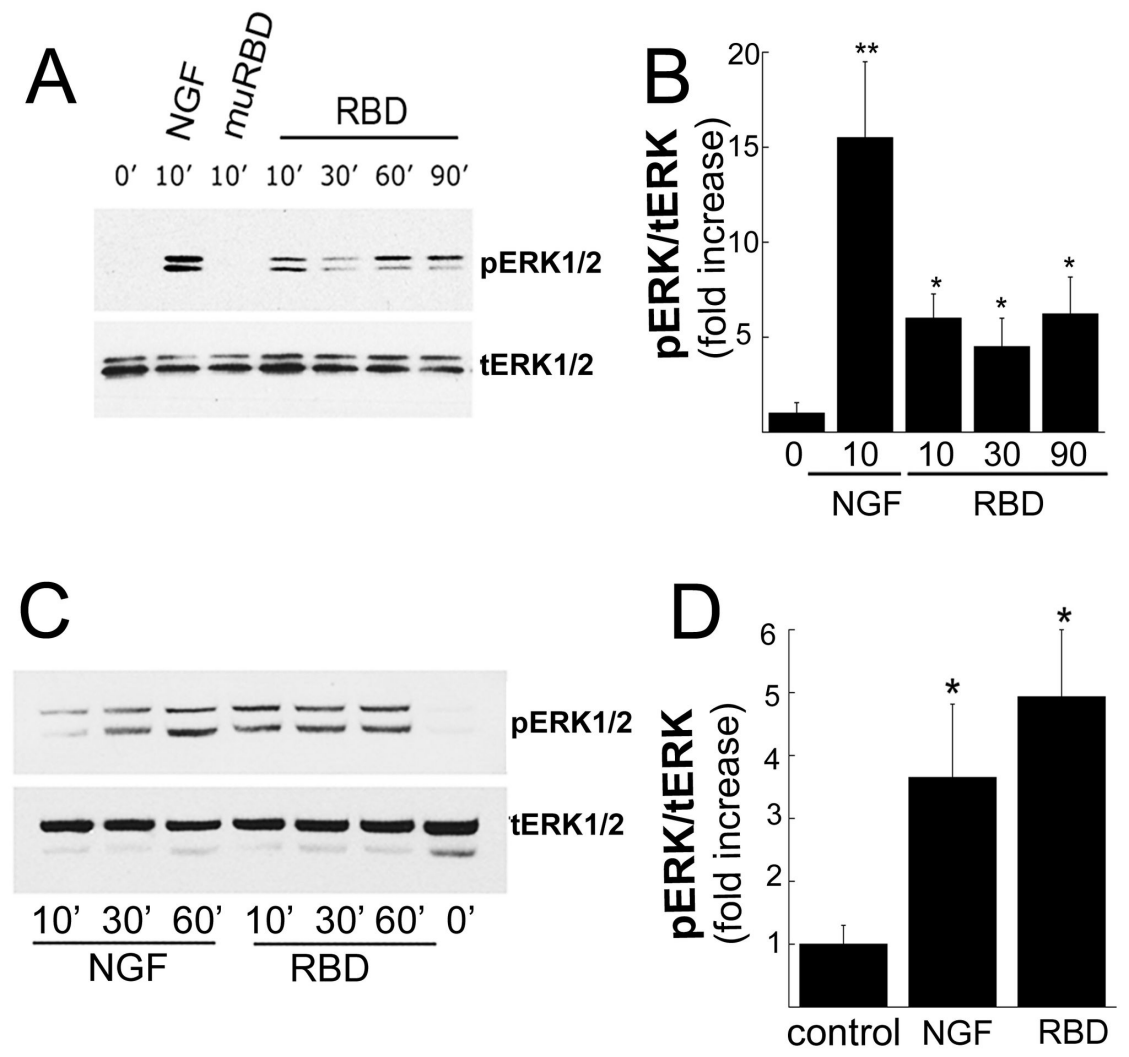
RBD induces LRP1-dependent and sustained ERK1/2 signaling in sensory neurons. **A**, Immunoblot analysis of ERK1/2 after treatment with RBD, muRBD, NGF or vehicle for 0-90 min in PC12 cells. **B**, Quantification of ERK1/2 by densitometry (n=3-6/group). Total ERK1/2 was used as a loading control (**p<0.01; *p<0.05). **C**, Immunoblot analysis of ERK1/2 after RBD, NGF or vehicle treatment for 0-60 min in primary embryonic sensory neurons. **D**, Quantification of ERK1/2 by densitometry (n=4/group). Total ERK1/2 was used as a loading control (**p<0.01; *p<0.05). Equal amounts of cellular protein (50 µg) were loaded into each lane and subjected to SDS-PAGE and electrotransferred to nitrocellulose for detection with specific antibodies. Primary cultures of DRG neurons were prepared from rat embryos day 15 (E15).

Previously, we showed that the RBD and other LRP1 ligands rapidly (within 5 min) induced activation of ERK1/2 [11,19]. To test whether LRP1 induces sustained (>10 to 90 min) activation of ERK1/2, we treated PC12 cells with RBD (100 nM) over time. RBD substantially increased sustained activation of ERK1/2 (p<0.05; Figure 1A,B). While the magnitude of activation was not as robust as NGF, the extent of activation was similar to NGF previously reported [28]. When we mutated RBD (muRBD) to convert lysine^1374^ and lysine^1370^ to alanine, LRP1 binding was precluded [11,17] and ERK1/2 activation did not occur, indicating that LRP1-dependent cell signaling was not operational. Thus, studies in PC12 cells demonstrated that RBD initiates LRP1-dependent cell signaling that is sustained.

**Figure 2:**
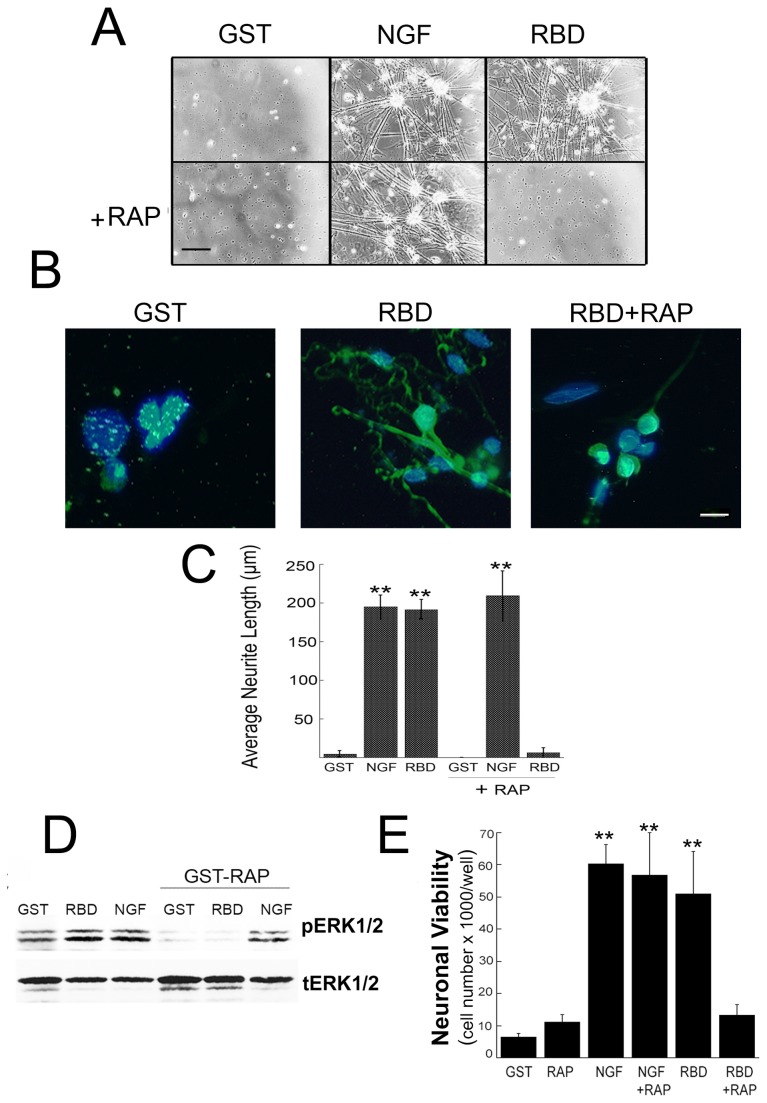
RBD promotes neurite sprouting in primary embryonic sensory neurons that is blocked by the LRP1 antagonist, GST-RAP. **A**, Phase-contrast images of DRG neurons cultured on collagen using an inverted microscope. DRG neurons were pretreated with or without GST-RAP (200 nM) for 30 min prior to the addition of RBD or NGF (50 ng/ml). Images represent n=6 independent studies. Scale bar, 50 µm. **B**, Immunolabeling of primary embryonic neurons with β neuronal class II tubulin (Tuj1; green) and Dapi (blue). Scale bar, 15 µm. **C**, Quantification of neurite sprouting in DRG neurons. Data are expressed as mean ± SEM (n=4 independent studies). In each study, 3 replicate wells were used to measure a minimum of 30 axonal extensions. **p<0.01 compared with respective GST or RAP controls. **D**, Immunoblot analysis of ERK1/2 in GST-RAP-treated DRG neurons subsequently stimulated with RBD or NGF for 30 min. The blot represents 2 independent experiments. **E**, Quantification of neuronal viability after two weeks in primary culture. Data are expressed as mean ± SEM (n=3) (**p<0.01).

We next examined effects of RBD in cultures of primary embryonic DRG neurons. After 2 weeks of culture in NGF and deoxyuridine to eliminate other proliferating cells [22], the remaining neurons exhibited extensive neurite outgrowth (Figure 2A,B). These neurons were then placed in serum free media for 1 h prior to cell signaling experiments. Neurons were then treated with RBD (100 nM) or NGF-β (50 ng/ml). RBD induced robust and sustained (>1 h) activation of ERK1/2 that was similar in extent and magnitude to NGF-β (p<0.05; Figure 1C,D).

### LRP1-dependent cell signaling promotes embryonic sensory neurite outgrowth

Next, we tested whether RBD promotes neurite outgrowth in a LRP1 dependent manner. Primary embryonic sensory neurons were cultured for 2 weeks with RBD, NGF or the negative control, GST. In some cases, cells were pretreated with RAP, which precludes binding of LRP1 ligands and blocks LRP1-dependent cell signaling [6,11]. NGF and RBD treatment resulted in the presence of viable sensory neurons that exhibited abundant neurite extension (Figure 2A,B). Quantitation demonstrated that NGF and RBD induced more than 40-fold increases in neurite outgrowth compared to GST controls (p<0.01; Figure 2C). Furthermore, the magnitude of increase in neurite outgrowth induced by RBD was similar to that observed by NGF (p<0.01; Figure 2C). In contrast, inhibition of LRP1 binding by RAP, the LRP1 antagonist, blocked neurite sprouting (Figure 2A-C). RAP completely blocked RBD-induced increases in neurite growth, but had no effect on neurite outgrowth in the absence of RBD. RAP had no effect on NGF-induced neurite outgrowth. To confirm that LRP1 was necessary for RBD-induced cell signaling in cultured embryonic DRG neurons, we pretreated neurons with the LRP1 antagonist, GST-RAP. GST-RAP completely blocked the ability of the LRP1 ligands to activate ERK1/2; however, cell signaling in response to NGF was unaffected (Figure 2D).

### LRP1 promotes survival in embryonic sensory neurons

**Figure 3:**
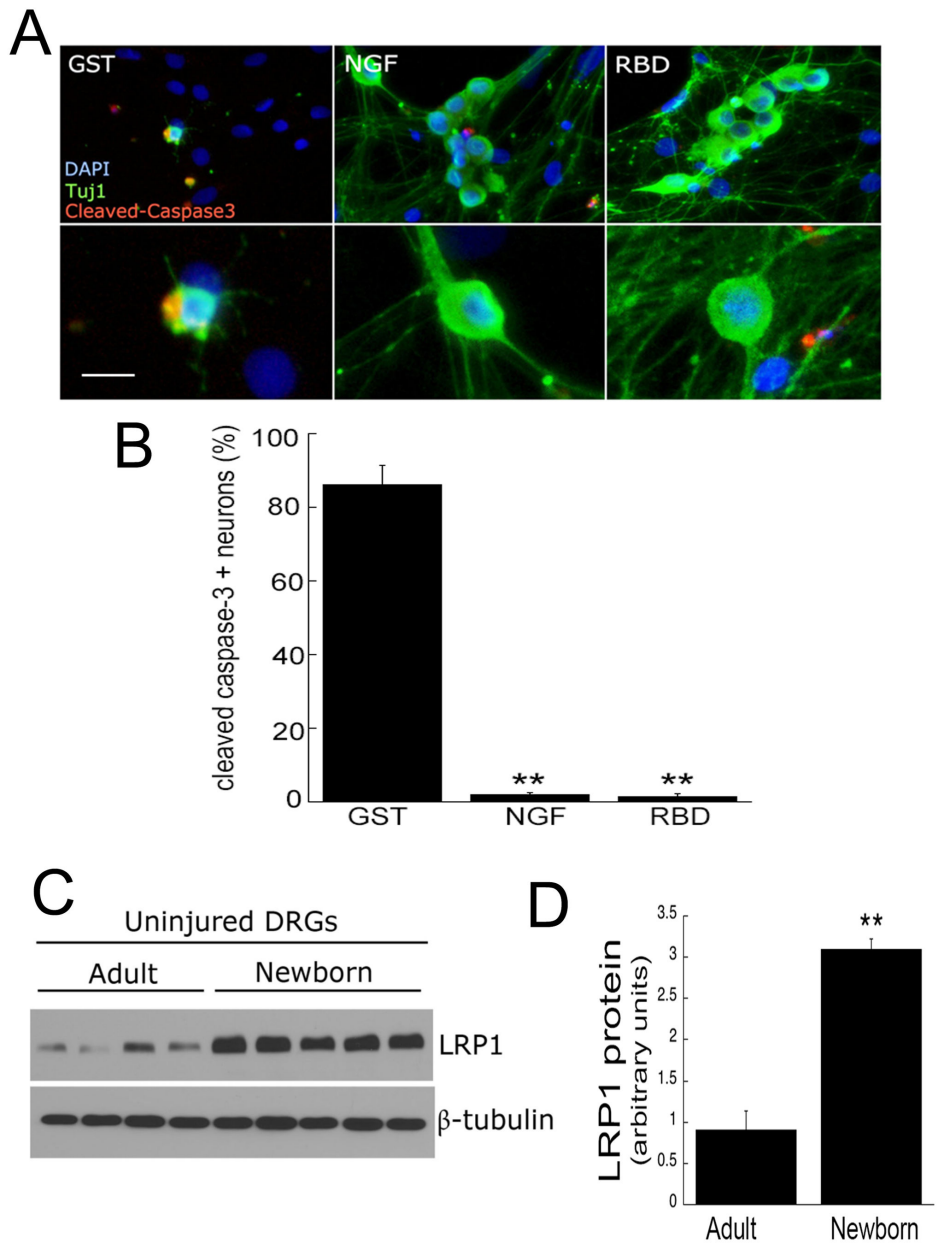
LRP1 promotes survival in primary embryonic sensory neurons and is abundantly expressed in neonatal dorsal root ganglia (DRGs). **A**, Immunofluorescence microscopy of cleaved caspase-3 (red) and β-neuronal class III tubulin (Tuj1; green) in primary DRG neurons cultured for 2 weeks. Scale bar, 12 µm. Cell nuclei are identified by Dapi (blue). Images represent n=3 independent replicates. **B**, Quantification of cleaved caspase-3 positive neurons. Neurons in random fields within each treatment group were counted (n=3 independent experiments). **C**, Immunoblot analysis of LRP1 (85 kDa) in uninjured adult (8 weeks) or neonatal (1 day) rat DRGs. DRGs were solubilized in RIPA buffer supplemented with sodium orthovanadate and proteinase inhibitors. β tubulin was used as a loading control. Equal amounts of cellular protein (20 µg) were loaded into each lane and subjected to SDS-PAGE and electrotransferred to nitrocellulose for detection with specific antibodies. Each lane represents an individual rat. **D**, Quantification of LRP1 by densitometry. Data are expressed as mean ± SEM (n=5-7 rats), **p < 0.01 compared with respective GST.

The function of LRP1 ligands such as RBD or tPA as potent activators of neuronal cell signalling raised the hypothesis that LRP1 may function as a pro-survival receptor for developing sensory neurons. Embryonic sensory neurons are dependent on neurotrophins for development prior to and when axons innervate their targets *in vivo* [3] and for survival during their first week of culture [29]. Thus, primary cultured embryonic sensory neurons are an excellent model to test whether RBD can facilitate their survival in the absence of NGF. Primary DRG cells were treated with GST (100 nM), RBD (100 nM) or NGF (50 ng/ml) with and without RAP, immediately after dissection and every other day. After two weeks in culture, DRG neurons were counted by Trypan blue exclusion. NGF and RBD treated neurons showed robust viability compared to GST, RAP or RBD+RAP treatments (p<0.01; Figure 2E). Next, DRG neurons were immunolabeled with β-neuronal tubulin to identify neurons, and cleaved caspase-3 to identify cells undergoing apoptosis. Neurons treated with RBD or NGF for one week were viable and showed almost no immunoreactivity for cleaved caspase-3 (p<0.01; Figure 3A,B). In contrast, sensory neurons treated with GST were immunolabeled for cleaved caspase-3. In some cases, neighboring non-neuronal cells were immunolabeled with cleaved caspase-3; this is likely due to deoxyuridine treatment that kills proliferating cells. Thus, RBD promotes the survival of primary embryonic sensory neurons *in vitro*.

The NGF receptor, TrkA, is expressed in approximately 90% of rodent DRG neurons immediately prior to birth and their proportion drops to less than 40% in adult DRGs [30,31]. We hypothesized that LRP1 levels may also be developmentally regulated given its putative role in survival signalling [7,14]. Using an antibody that recognizes the transmembrane small chain LRP1 (85 kDa), we measured LRP1 levels in DRG lysates from both newborn and adult DRGs by immunoblot analysis. LRP1 levels were 3-4-fold greater in post-natal compared to adult DRGs (p<0.01; Figure 3C,D).

### Src-family kinases (SFKs) are activated and required for LRP1-induced neurite sprouting

**Figure 4:**
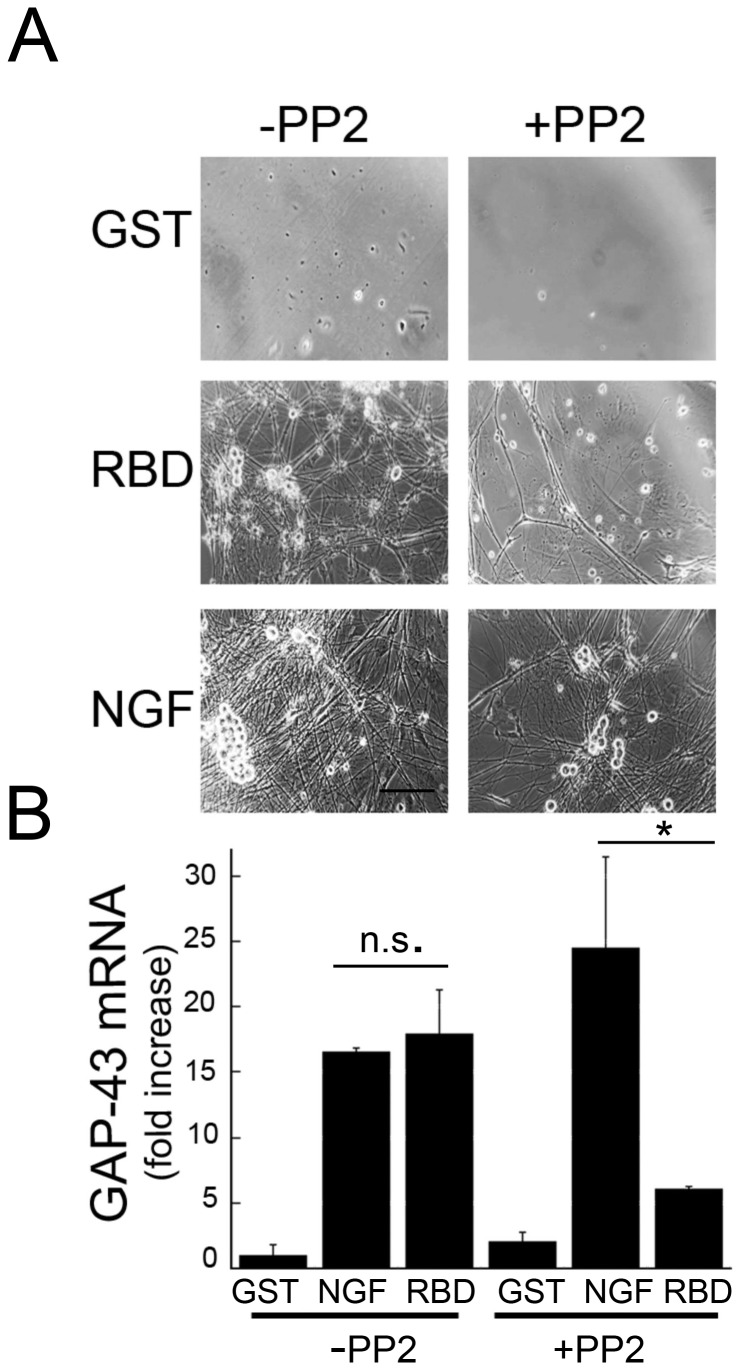
RBD-induced neurite sprouting is blocked in primary sensory neurons pre-treated with a Src-family kinase inhibitor, PP2. **A**, Phase-contrast images of cultured sensory neurons after 2 weeks of treatment. Note the reduction of sprouting in RBD (100 nM) treated cultures, but not NGF (50 ng/ml) treated cultures with treatment of PP2 (1 µM) for 30 min prior to the addition of factors. Scale bar, 50 µm. **B**, Levels of GAP-43 mRNA in primary cultures treated with RBD (100 nM) or NGF (50 ng/ml) for 2 weeks, with or without PP2 treatment. *p<0.05 (n=3-4 independent experiments).

Because LRP1 transactivates Trk receptors in a Src-family kinase (SFK) dependent-manner in PC12 cells [12] and embryonic sensory neurons are initially dependent upon Trk activation for survival [3], we tested whether LRP1 ligands promote neurite sprouting in developing neurons through an SFK-mediated mechanism. We used growth-associated protein-43 (GAP-43) as a quantitative index of neurite sprouting. GAP-43 is a highly expressed protein in neuronal growth cones during development that is used as a marker of growth or plasticity in regenerating axons [32]. Primary embryonic neurons were cultured with a selective pharmacological inhibitor of intracellular SFK (PP2), with and without RBD or NGF for 2 weeks. Both RBD and NGF manifested neurite sprouting (Figure 4A) and significant increases in GAP-43 mRNA expression (>17-fold) compared to GST controls (p<0.01; Figure 4B). The extent of RBD’s neurotrophic effects was similar to NGF. However, in the presence of PP2, neurite outgrowth and GAP-43 expression were significantly reduced in RBD treated neurons, although some sprouting and GAP-43 expression remained. In contrast, NGF treated neurons still had groups of viable neurons, extensive neurite outgrowth and increased GAP-43 expression in the presence of PP2 (>20-fold). Collectively, these results indicate that LRP1 ligands promote embryonic sensory neurite extension via an SFK-mediated pathway and further supports a model in which LRP1 ligands indirectly activate Trk receptors through an intracellular SFK-dependent transactivation pathway

**Figure 5:**
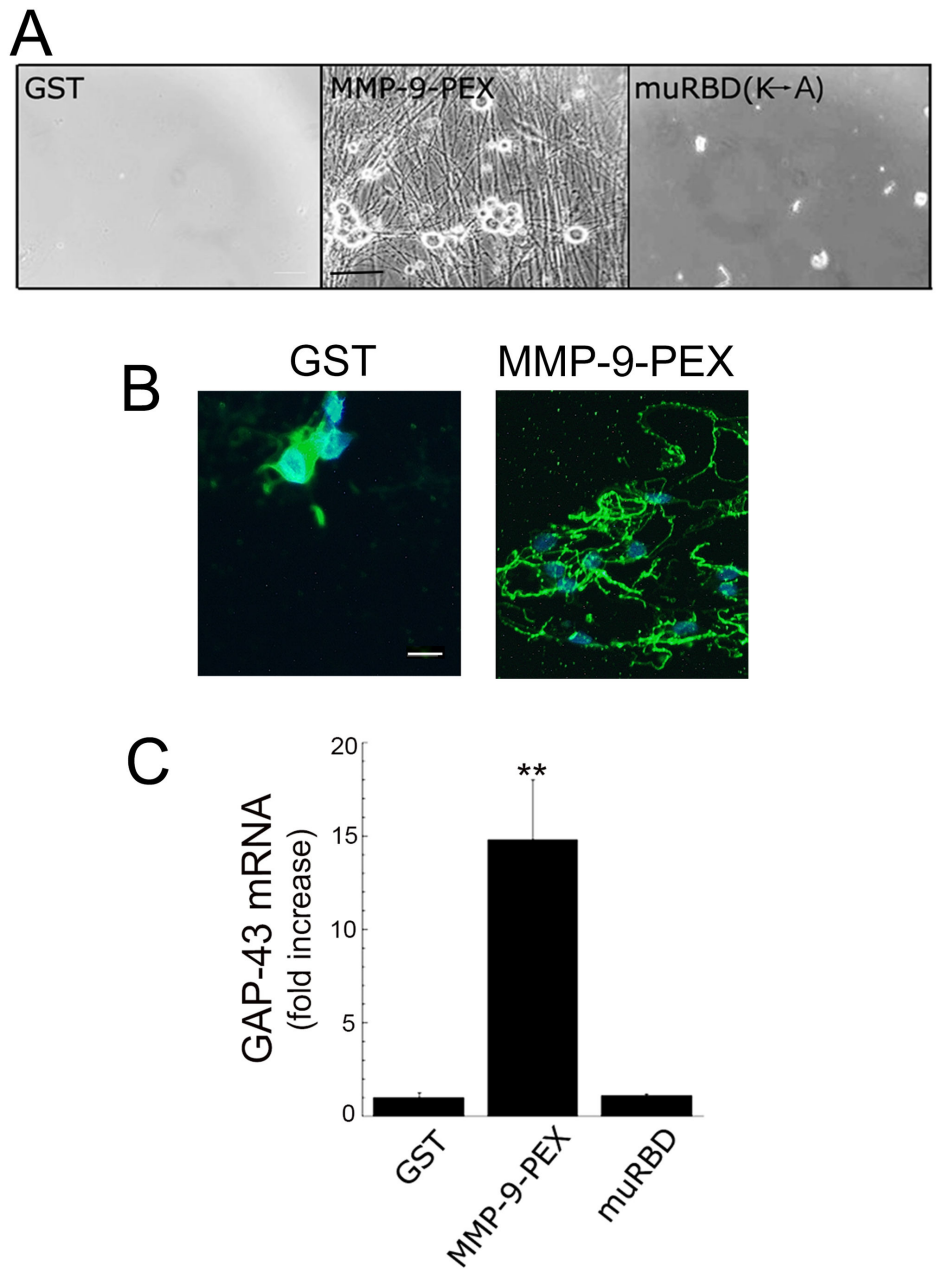
Structurally diverse LRP1 ligands such as MMP-9-PEX, but not mutated RBD (muRBD), promoted sensory neurite extensions. **A**, Phase contrast images of cultured sensory neurons treated with the hemopexin domain of MMP-9 (MMP-9-PEX; 100 nM), mutated RBD (muRBD; lysine residues 1370 and 1374 that are essential for LRP1 binding are replaced by alanine; 100 nM) or GST (100 nM). Scale bar, 50 µm. **B**, Immunofluorescence microscopy of β-neuronal class III tubulin (Tuj1; green) in primary DRG neurons cultured for 2 weeks in MMP-9-PEX or GST. Scale bar, 10 µm. Cell nuclei are identified by Dapi (blue). Images represent n=2 independent replicates. **C**, GAP-43 mRNA levels in cultured sensory neurons treated with GST, MMP-9-PEX or muRBD every other day for 2 weeks. Results are compared to vehicle-treated control cultures. Data are expressed as mean±SEM (n=2-4). **p < 0.01 compared with controls.

As a second approach to study the role of LRP1 in neurite outgrowth, we examined a structurally distinct LRP1 ligand, the hemopexin domain of matrix metalloproteinase-9 (MMP-9-PEX). Previously, we have shown that MMP-9-PEX activates cell signaling in PC12 cells and Schwann cells by binding to LRP1 [12,19]. After 2 weeks in culture, MMP-9-PEX –treated neurons were viable in the absence of neurotrophins, and induced robust neurite outgrowth that was quantified by a 15-fold increase in expression of GAP-43 compared to GST (p<0.01; Figure 5A-C). We also treated cultured neurons with muRBD (100 nM), a mutated LRP1 ligand that is unable to bind LRP1 [11,16] and failed to induce activation of ERK1/2 (Figure 1). In primary embryonic sensory neurons, muRBD also failed to promote neuronal survival, outgrowth and expression of GAP-43 (Figure 5).

### Embryonic sensory neurons treated with LRP1 agonists are receptive to Schwann cells

**Figure 6:**
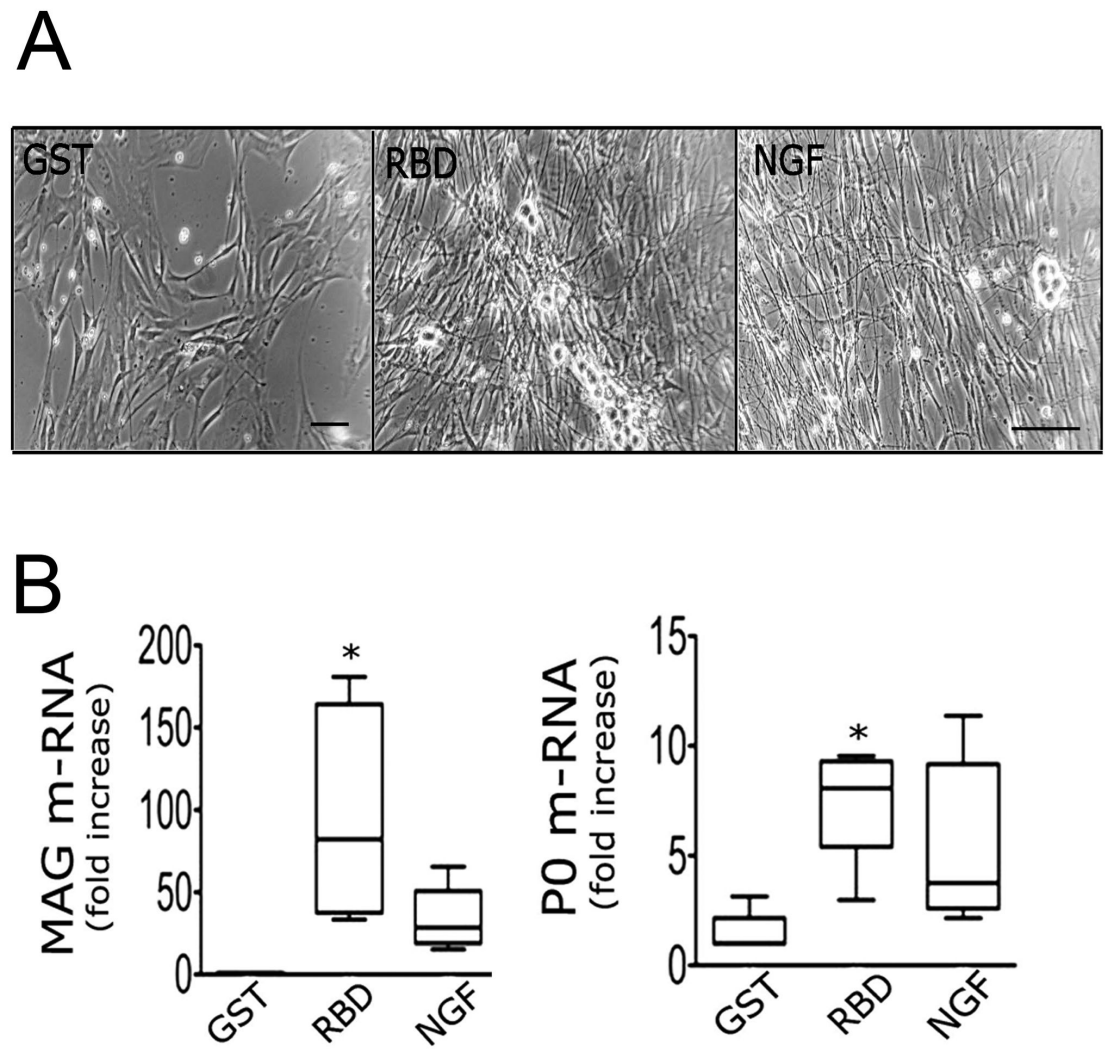
Axonal Receptivity to myelination by Schwann cells after treatment with LRP1 ligands. **A**, Phase-contrast images of primary sensory neurons treated with NGF (50 ng/ml) or RBD (100 nM) for 2 weeks and now with Schwann cells for 48 hours. Scale bar, 50 µm. Images represent n=3 independent replicates with internal duplicates; **B**, Levels of the non-compact myelin marker, MAG and **C**, compact myelin marker, P0 mRNA in Schwann cells after 48 hours of co-culture with RBD or NGF treated DRG neurons. Data are expressed as mean ± SEM. *p < 0.01 compared with GST (n=3 independent experiments).

Because axons may dictate whether or not they will become myelinated in the PNS, and that NGF is a potent regulator of these axonal signals [33], we investigated whether cultured DRG neurons treated with LRP1 agonists are receptive to Schwann cells. First, we established neurite-bearing cultures of embryonic sensory neurons by treating neurons with RBD (100 nM), NGF (50 ng/ml) or GST (100 nM; negative control) for 2 weeks. Both RBD and NGF manifested neurite outgrowth as shown (Figures 2-5). Equal numbers of primary Schwann cells were subsequently seeded onto neurons and co-cultured for 2 days without further treatment. Co-cultures treated with RBD or NGF exhibited organized, elongated spindle shaped Schwann cells, as anticipated (Figure 6A). Next, we measured mRNA for two significant proteins that are present in peripheral myelin: P0, a major component of structural myelin; and myelin-associated glycoprotein (MAG), an essential component of non-compact myelin. RBD significantly increased both MAG and P0 mRNA compared to GST controls after 48 h of co-culture (Figure 6B; p<0.05). The magnitude of RBD-induced myelin gene expression was similar or greater than NGF treatment, indicating that RBD induced axon signals are capable of promoting receptivity to myelination by Schwann cells.

## Discussion

Accumulating evidence indicates that LRP1 activation plays a role in the survival of glia and some CNS neuronal populations [7,14]. However, the relevance of this activity in regulating growth, differentiation and survival of developing peripheral neurons is unknown. Herein, we implicate LRP1 as a novel neurotrophic receptor for embryonic sensory neurons. These results provide the first evidence that a new class of receptors, the LDL receptor gene family, regulate survival and growth of developing sensory neurons. Specifically, LRP1-dependent cell signaling emerges as a novel mechanism underlying neurotrophic activity during development.

During early stages of development, many sensory neurons of the dorsal root ganglion switch their survival requirements from one neurotrophin to another [34] and are ultimately dependent on NGF prior to birth. NGF is a potent neurotrophic factor [35] that binds primarily to the receptor tyrosine kinase (RTK), TrkA [36]. Ligand binding induces dimerization of the receptor and recruits adapter proteins (Shc and Grb2) as well as enzymes (PI3K/Akt, PLCg) that contribute to sustained Ras/ERK1/2 signaling and neurite outgrowth [37,38,39]. Herein, we show for the first time that LRP1-dependent cell signaling also mediates sustained ERK1/2 activation in PC12 cells and primary embryonic sensory neurons, independently of NGF. Sustained ERK1/2 signaling is associated with neuronal differentiation [40,41] and indeed LRP1-dependent cell signaling induced robust neurite extension in primary sensory neurons.

Mechanistically, we propose two possibilities for LRP1 activity: 1) Levels of LRP1 in embryonic sensory neurons are abundant, and therefore activate sustained ERK1/2 signaling and differentiation. Previously, it has been shown that there is no receptor specific pathway of differentiation, however, the number of RTKs located on the cell surface appear to directly regulate the duration of ERK1/2 activation [40]. For example, in PC12 cells, stimulation of the endogenous EGF receptor leads to transient activation of ERK1/2 without differentiation, while overexpression of EGF leads to EGF-dependent differentiation and sustained activation of ERKs [38]. Moreover, when PDGF receptors (which are not endogenously expressed by PC12 cells) are overexpressed, PDGF induces sustained activation of ERKs and neurite outgrowth [42]. Conversely, if the number of TrkA receptors is reduced, NGF is no longer able to induce differentiation or sustained ERK1/2 signaling. Although LRP1 is not a RTK, its cytoplasmic tail can become phosphorylated and recruit adapter proteins [43] and, thus, elevated levels of LRP1 in neonatal DRGs represents a mechanism by which LRP1 ligands can activate and support sustained ERK1/2 signaling and differentiation; 2) LRP1 transactivates Trk receptors in embryonic sensory neurons through a SFK-dependent pathway. Previously, we have demonstrated that LRP1 transactivates Trk via Src in PC12 cells and cerebellar granular neurons [12]. Src is associated with neuronal differentiation in PC12 cells [44] and participates in cooperative signal amplification from other ligand receptor systems. This cooperation may provide more continuous activation of Trk and downstream signaling of ERK1/2. Concomitant activation of RTKs and LRP1 leads to sustained activation of ERK1/2 in fibrosarcoma cells [45]. Accordingly, we show that the pharmacological inhibitor of Src, PP2, reduces the ability of LRP1 ligands to extend neurites, but still survive, suggesting that LRP1-dependent cell signaling pathways of differentiation are SFK-mediated in embryonic sensory neurons.

Previously, it was reported that LRP1 ligands may bind directly to TrkA independently of LRP1 and antagonize TrkA-initiated signaling [46], yet our results in primary embryonic sensory neurons support another model. We show that structurally distinct LRP1 ligands, RBD and MMP-9-PEX, induce neurite outgrowth equivalently, which was blocked by RAP, a competitive antagonist of LRP1 ligand binding. Furthermore, a mutated form of RBD that precludes LRP1 binding was ineffective in inducing ERK1/2 activation or neurite sprouting. These findings provide evidence that LRP1 ligands have direct interaction with LRP1 and lead to signaling activity. Direct ligand binding to LRP1 and subsequent cell signaling activity has been shown in primary Schwann cells [11,19].

We also identified LRP1 as a potent survival factor for primary embryonic sensory neurons. Without LRP1 ligands or NGF, neurons did not survive as evidenced by trypan blue exclusion, cleaved caspase-3 and morphological assessment. The effectiveness of LRP1 ligands was similar to NGF. Yet, why do developing neurons need multiple signaling mechanisms to promote survival? One possibility is that dependence on survival signaling to control cell numbers is complex in vertebrate systems. Cells are forced to compete with one another for limiting amounts of survival factors. In the developing peripheral nervous system, competition for target derived survival signals is operational and plays an important role in matching the numbers of presynaptic and postsynaptic cells during both evolution and development [1,47,48]. Competing for limiting amounts of multiple survival factors could more finely select cells that establish optimal contacts during development, enhancing competitive mechanisms. The most competitive cells may be those with the most receptors for survival or those with the most efficient signal transduction [49]. LRP1 may provide adaptive strategies for neurotrophic activity when target derived growth factors are limited or “switching” [34]. Accordingly, LRP1 antagonizes the unfolded protein response (UPR) or ER stress pathways that ultimately lead to death if not countered [50].

The demonstration of potent LRP1 signaling effects on neuronal survival and process outgrowth during development may have implications for axonal plasticity and regeneration in adulthood. Indeed, studies in progress suggest that the LRP1 ligands RBD and MMP-9-PEX enhance sensory axonal sprouting and regeneration after spinal cord injury [15], highlighting the importance and relevance of this novel signaling mechanism in nervous system development and function.
